# Applications of a Specialty Bicuspid Aortic Valve Program: Clinical Continuity and Translational Collaboration

**DOI:** 10.3390/jcm9051354

**Published:** 2020-05-05

**Authors:** Erin E. Crawford, Patrick M. McCarthy, S. Chris Malaisrie, Jyothy J. Puthumana, Joshua D. Robinson, Michael Markl, Menghan Liu, Adin-Cristian Andrei, David G. Guzzardi, Jane Kruse, Paul W. M. Fedak

**Affiliations:** 1 Division of Cardiac Surgery, Northwestern Medicine, Chicago, IL 60611, USA; ecrawfor@nm.org (E.E.C.); chris.malaisrie@nm.org (S.C.M.); menghan.liu@northwestern.edu (M.L.); JKruse@nm.org (J.K.); 2 Department of Surgery, Feinberg School of Medicine, Northwestern University, Chicago, IL 60611, USA; 3 Division of Cardiology, Northwestern Medicine, Chicago, IL 60611, USA; jyothy.puthumana@nm.org; 4 Department of Internal Medicine, Feinberg School of Medicine, Northwestern University, Chicago, IL 60611, USA; 5 Division of Cardiology, Ann & Robert H Lurie Children’s Hospital of Chicago, Chicago, IL 60611, USA; jdrobinson@luriechildrens.org; 6 Department of Radiology, Feinberg School of Medicine, Northwestern University, Chicago, IL 60611, USA; mmarkl@northwestern.edu; 7 Department of Pediatrics, Feinberg School of Medicine, Northwestern University, Chicago, IL 60611, USA; 8 McCormick School of Engineering, Evanston, IL 60208, USA; 9 Department of Preventive Medicine, Feinberg School of Medicine, Northwestern University, Chicago, IL 60611, USA; Adin-Cristian.Andrei@nm.org; 10 Section of Cardiac Surgery, Department of Cardiac Sciences, Cumming School of Medicine, University of Calgary, Calgary, AB T2N 4N1, Canada; dgguzzardi@gmail.com; 11 Libin Cardiovascular Institute, Calgary, AB T2N 4N1, Canada

**Keywords:** bicuspid aortic valve, aortic aneurysm, congenital heart disease, 4D MRI

## Abstract

Bicuspid aortic valve (BAV) is a common congenital heart diagnosis and is associated with aortopathy. Current guidelines for aortic resection have been validated but are based on aortic diameter, which is insufficient to predict acute aortic events. Clinical and translational collaboration is necessary to identify biomarkers that can individualize the timing of prophylactic surgery for BAV aortopathy. We describe our multidisciplinary BAV program, including research protocols aimed at biomarker discovery and results from our longitudinal clinical registry. From 2012–2018, 887 patients enrolled in our clinical BAV registry with the option to undergo four dimensional flow cardiovascular magnetic resonance imaging (4D flow CMR) and donate serum plasma or tissue samples. Of 887 patients, 388 (44%) had an elective BAV-related procedure after initial presentation, while 499 (56%) continued with medical management. Of medical patients, 44 (9%) had elective surgery after 2.3 ± 1.4 years. Surgery patients’ biobank donations include 198 (46%) aorta, 374 (86%) aortic valve, and 314 (73%) plasma samples. The 4D flow CMR was completed for 215 (50%) surgery patients and 243 (49%) medical patients. Patients with BAV aortopathy can be safely followed by a multidisciplinary team to detect indications for surgery. Paired tissue and hemodynamic analysis holds opportunity for biomarker development in BAV aortopathy.

## 1. Introduction

Bicuspid aortic valve (BAV) is a common congenital diagnosis involving both the aortic valve and aorta, and is often categorized with connective tissue disorders such as Marfan and Turner syndromes. In adults, BAV typically presents as progressive valve deterioration through aortic stenosis (AS), insufficiency (AI), or infective endocarditis (IE). These valvular complications are accompanied by aortopathy in up to 42% of cases, including dilatation of the aortic root, ascending aorta, arch, or coarctation [1]. Additionally, both BAV and associated aortopathy are heritable among first-degree relatives (FDR), warranting echocardiographic screening for all FDR of a BAV proband [2,3].

Though BAV is the most common congenital heart diagnosis, the malignancy of BAV-associated aortopathy is challenging to define. Risk-stratification is further complicated by the heterogeneity of BAV—previous studies show patients’ phenotypes and risks differ across age groups, sexes, and clinical settings [4,5,6]. We developed a specialty BAV program with the goals to enhance clinical continuity for patients and families, and to integrate a longitudinal clinical registry with translational research efforts aimed at developing additional biomarkers for BAV aortopathy. This multidisciplinary approach is similar to the ‘Heart Team’ clinical model for severe aortic stenosis, complex coronary artery disease, and advanced heart failure management [7,8,9,10]. To our knowledge, this is a unique approach to BAV management. We aim to share our experience by describing patients’ characteristics, treatment courses, and volume of translational specimens collected to date.

## 2. Materials and Methods

### 2.1. BAV Registry

From 2012–2018, 887 patients with BAV enrolled in our single-center registry after presenting to our referral cardiology or cardiac surgery clinic. Patients were assigned to a surgical or medical group based on clinical recommendations at time of presentation. We abstracted baseline and follow up clinical data from the medical record annually, including family history and imaging variables from echocardiography and cardiac magnetic resonance imaging (CMR). Baseline aortic diameter and valve morphology data were abstracted from clinical CMR reports; if a baseline CMR was not available, echocardiography values were used. Beginning in 2017, patients received annual follow up surveys to capture updates in social and family histories, including new BAV screening results of first-degree relatives.

### 2.2. Adolescent Transition Clinic

Once a month, we hold a multidisciplinary transition clinic for 16–26 year old patients with BAV. A team of adult and pediatric providers gradually transfer the patient to the adult cardiology clinic and coordinate FDR screening as needed. These transitioning patients may enroll in the pediatric arm of the aforementioned observational registry. To compute the aortic diameter Z-score in the adolescent population, aortic measurements were normalized using the Boston echocardiography Z-score methods from 2013 and, more recently, 2017.

### 2.3. Statistical Methods

Continuous variables were summarized using means/standard deviations, for discrete ones we presented counts and percentages. Group comparison were based on one-way ANOVA for continuous variables and the Wilcoxon rank sum test or Fisher’s exact test (when <5 cell counts) for discrete variables. Overall survival from the time of surgery (for surgical patients) or consent (for medical patients) was estimated using the Kaplan-Meier estimator. A comparison of overall survivorship since surgery among the surgical aortic valve replacement (SAVR), SAVR with aneurysm resection (SAVR + AN) and transcatheter aortic valve replacement (TAVR) groups was based on the log-rank test. Two-sided *p*-values less than 5% were considered statistically significant, and there were no multiplicity adjustments. All analyses were performed in SAS v 9.4 (SAS Institute: Cary, NC, USA).

### 2.4. Translational Research Enrollment

Adult patients had the option to undergo the procedure (4D-CMR) for the assessment of BAV-mediated alterations in aortic hemodynamics (flow patterns, wall shear stress) and donate serum plasma samples; those undergoing surgery could opt to donate aortic valve or aorta tissue samples, which were resected per clinical guidelines.

## 3. Results

### 3.1. Clinical Demographics

Of 887 patients (Table 1), 388 (44%) had an elective BAV-related procedure after initial presentation, while 499 (56%) continued with medical management. The average follow up period was 2.9 ± 1.8 years (range of 7.0 days to 7.0 years). Forty four (9%) medical patients underwent elective surgery after 2.3 ± 1.4 years. Five-year survival was 95.6% in the surgical group, 99.8% in the medical group, and 96.7% in the medical-to-surgical group (*p* = 0.03).

The age range of all patients in our adult cohort was 20 to 89 years. None of our medically-followed patients had an aortic dissection during the follow up period. Endocarditis was a surgical indication for 10 (2%) patients in the surgical group and none in the medical to surgical group. Three (1%) patients in the medical group have a history of chronic endocarditis, managed medically.

A total of 107 (15%) patients report a family history of BAV. Of these, 94 (13%) patients had a known family history of BAV at baseline and an additional 13 (2%) discovered one or more family members with BAV through follow up screening. Family screening of 250 FDR (from 130 families) identified 16 new FDR with BAV, 11 of whom subsequently enrolled in our BAV program.

### 3.2. Surgical and Transcatheter Interventions

**Figure 1:**
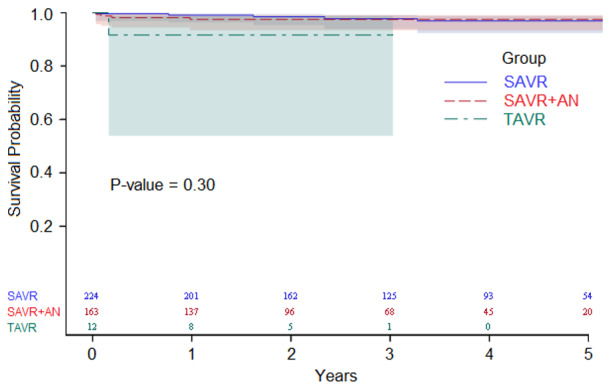
Overall survival estimates in the surgical or transcatheter interventions group.

The total 432 BAV procedures included 224 (52%) SAVR, 163 (38%) SAVR + AN, 18 (4%) aortic valve repairs (9 (50%) with AN), 15 (3%) valve-sparing root repairs; and 12 (3%) TAVR (Table 2). Bioprosthetic valves were used in 364 (91%) SAVR and SAVR + AN. Hemiarch was performed for 70 (16%) surgeries. Thirty-day survival for all procedures was 99%, with no significant differences in overall survival in follow up (*p* = 0.30, Figure 1).

One patient had a previous balloon aortic valvuloplasty, six (1.3%) had a history of aortic coarctation (none with previous repair), and nine (2%) had a previous congenital surgery or procedure.

### 3.3. Adolescent Transition Clinic

Since 2016, 45 (54%) of 83 adolescent patients from our transition clinic enrolled in our registry, 17 (38%) demonstrate moderate or more AS, moderate or more AI, or an ascending aortic diameter with Z-score greater than 4 by echocardiogram (Table 3). Three (7%) patients have a history of aortic coarctation, two of which are now post-repair. One patient underwent successful aortic valve and ascending aorta replacement at our pediatric center; preoperatively, he had symptomatic moderate-severe aortic stenosis (peak velocity 4.0 m/s) and severe aortic regurgitation (regurgitant fraction 31–37% with left ventricular volume indexed to 130 mL/m^2^). His ascending aorta measured 45 mm with a Z-score of +5.76.

Sixteen FDR (from eight families) were referred to our adult center for family screening, leading to detection of BAV in one (6%) family member, who established care in our adult BAV clinic. In surveying patient encounters across all ages, four pediatric patients have transitioned to the adult cardiology clinic since their initial visit in the transition clinic.

### 3.4. Translational Research Enrollment

**Figure 2:**
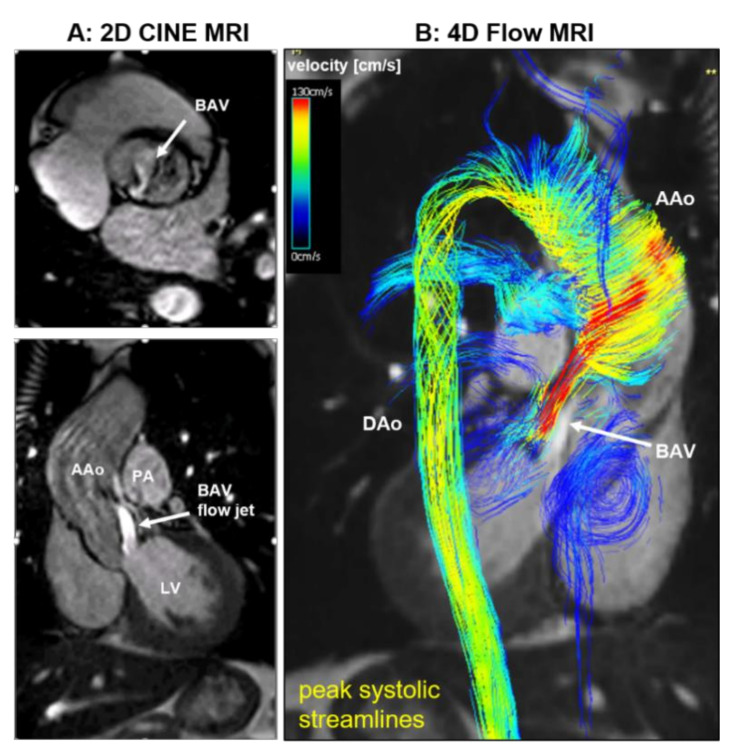
The 2D CINE (**A**) and 4D flow cardiovascular magnetic resonance (CMR) (**B**) in a 32-year old patient with bicuspid aortic valve (BAV, right-noncoronary (R-N) valve fusion pattern), aortic dilatation (Sinus of Valsalva/Mid Ascending Aorta diameter = 42/45 mm), moderate aortic valve stenosis (aortic valve area = 1.5 cm^2^). A: 2D CINE MRI during peak systole shows limited BAV opening (top) and the associated outflow jet (bottom) B: 3D streamline visualization of systolic blood flow in the thoracic aortic as assessed by 4D flow CMR and shows a marked high-velocity (red color) valve outflow jet directed toward the anterior wall of the ascending aorta (AAo). Note the formation of a complex helix flow pattern in the entire AAo. In addition, 4D flow CMR provides full volumetric coverage of the thoracic aorta and flexible retrospective quantification of peak systolic velocities at multiple locations in the thoracic aorta. LV = left ventricle, PA = pulmonary artery, DAo = descending aorta.

Of all surgery patients, 198 (46%) donated aortic tissue samples, 374 (86%) donated aortic valve samples, and 314 (73%) donated plasma samples (vs. 84% of the medical group). The 4D flow CMR (Figure 2) was completed for 215 (50%) surgery patients and 243 (49%) of all medical patients.

## 4. Discussion

### 4.1. Specialty BAV Program in the Era of the Heart Team

Multidisciplinary collaboration between cardiologists, cardiac surgeons, nurse coordinators, and imaging specialists is an increasingly common approach to managing cardiac diagnoses. The ‘Heart Team’ was coined to represent variations of this shared decision-making model for treatment of severe aortic stenosis, complex coronary artery disease, and advanced heart failure [8,9,10,11]. Our BAV program functions similarly to the Heart Team, but with additional emphasis on translational research and intergenerational screening. Other centers treating BAV aortopathy may find all or parts of our framework helpful, or better yet, expand on it with their own strengths or areas of expertise.

### 4.2. Clinical Demographics

Data from large groups of patients with BAV suggest excellent long-term outcomes with very low risk of catastrophic events and survival similar to a non-BAV cohort. Most patients are referred to our BAV program based on clinical findings suggestive of significant AS or AI, or an incidental finding of aortopathy noted on imaging. The incidence of BAV endocarditis is 2% in contemporary BAV cohorts, which is similar to our experience and 16 times higher than the incidence of the general population [12]. Both transthoracic echocardiography (TTE) and transesophageal echocardiography (TEE) are critical in diagnosis and clinical decision-making for infective endocarditis [13,14].

We note that our series describes patients presenting to a referral center, with clinical courses and phenotypes that differ from those followed in the community setting [4,5,6]. While patients with valvular manifestations will eventually develop symptoms as the disease progresses, those with aortopathy as a sole manifestation may have a clinically silent course, underscoring the importance of serial aortic imaging strategy.

### 4.3. Imaging of the Aorta

Due to the asymmetric nature of aortopathy in BAV, recent BAV aortopathy guidelines recommend ECG-gated CT or MR angiography (CTA or MRA) as the gold standard imaging modality, due to their use of the double-oblique technique to measure aortic diameter perpendicular to the longitudinal axis of the aorta [15]. Additionally, CTA and MRA permit repeat measurements at precise locations along the aorta for consistent comparison between serial studies. If MRA or CTA are contraindicated for a patient, the aorta may be measured by TTE from leading edge-to-leading edge at end-diastole, which correlates best with CTA and MRA diastolic inner wall to inner wall measurement [15,16].

MRA is an attractive option for aortic imaging for BAV aortopathy as it minimizes contrast and radiation exposure for patients who tend to present at a young age and may require multiple studies before meeting a surgical endpoint [17,18]. Additionally, information about wall shear stress from 4D flow CMR analysis has the potential to reach diagnostic quality without the use of contrast [19].

If baseline root and tubular ascending diameters are normal, follow up monitoring can be performed every 3–5 years. This practice aligns with the guideline recommendations for annual imaging if the baseline root and/or tubular ascending diameters are dilated (40–49 mm), reducing frequency to every 2 years if no progression is noted between serial studies [15]. If root and/or tubular ascending diameters are 50–54 mm, annual or semiannual imaging are warranted. Aortopathy progression tends to be faster in patients with BAV versus those with trileaflet aortic valve (TAV): some studies report an average aortic growth rate of 1.9 mm/year in BAV versus 1.3 mm/year in patients with TAV [17] another study suggests an even slower growth rate of 0.4–0.6 mm/year [20].

The abdominal and pelvic aorta do not require monitoring in isolated BAV disease, unless a family history of abdominal or iliac aneurysms is present [21]. Although intracranial arterial aneurysms have been found more commonly in patients with BAV, there is no increased prevalence of intracranial aneurysm–related subarachnoid hemorrhages in BAV patients other than patients with coarctation; therefore, routine brain angiography is only recommended in patients with BAV and concomitant coarctation of the aorta [15,22,23].

### 4.4. Family Screening

Previous family screening studies report finding BAV in FDR of 9–11% of BAV index patients [2,24,25,26]. These were cross-sectional, single-center studies with consistent screening protocols and inter-reader validity. Our longitudinal series reports a slightly higher rate of BAV family history (15%), possibly because patients are surveyed annually for updates in family screening. This allows us to capture screening results from outside centers, overcoming logistical and geographical barriers to screening complete sets of FDR.

In addition to BAV heredity, FDR are more likely to have a dilated aorta, with or without concomitant BAV [2]. Nineteen (2%) patients in our program reported a family history of thoracic aortic aneurysm, two of which required aortic resection during the follow up period. Additionally, one patient reported a new family history of aortic dissection in an otherwise healthy family member with a TAV aneurysm.

Recent studies propose familial inheritance of a milder phenotype of BAV, marked by a smaller cusp fusion, the presence of aortic dilation, and atypical aortic blood flow and wall shear stress [25,27,28]. The smaller raphe in these family members is often missed by echocardiography—the standard imaging modality used for FDR screening—suggesting that familial clustering of BAV is underestimated.

### 4.5. Surgical and Transcatheter Interventions

The majority of patients facing surgery for BAV valvulopathy require valve replacement for aortic stenosis. Recently, TAVR has become an alternative to SAVR for BAV patients with AS. Outcomes for TAVR in highly selected BAV patients with AS have improved significantly with newer generation valves and is well accepted in patients with a high and intermediate surgical risk [29]. However, the higher rate of stroke, annular rupture, and need for permanent pacemakers remain concerns when considering TAVR for the more typical young BAV patient with low-surgical risk [30,31]. Randomized clinical trials for BAV patients with AS have not been performed, and until both safety and long-term efficacy are demonstrated, SAVR with or without aneurysm repair should be considered the gold standard therapy for these patients.

Guidelines recommend bioprosthetic valve replacement for patients 60 years and older, with the expectation that valve durability lasts 15–20 years in this age group [32]. Bioprosthetic valves are also an attractive option for patients who want to avoid managing Coumadin protocols for stroke prophylaxis after mechanical aortic valve replacement. Should the bioprosthetic valve degenerate later in life, it can be replaced through a transcatheter valve-in-valve procedure; this bypasses the need for a repeat sternotomy in an older and likely higher-risk patient [33]. These considerations and patient preference factor into our use of bioprosthetic valves in 91% of the SAVR cohort (Table 2: median age 58 ± 12 years). Our follow up period is short, however a contemporary study of patients aged 50–69 years suggests patients with mechanical or bioprosthetic aortic valve replacements show no significant difference in survival at 15 years [34].

Current guidelines recommend prophylactic aortic resection for diameters between 45 mm and 55 mm, depending on indications for concomitant valve intervention and risk factors for surgery [15]. For patients without valve disease, aortic resection may be postponed until the aorta reaches 55 mm. Risk factors such as family history of thoracic aortic dissection or aortic growth rate > 5.0 mm per year merit aortic resection at a smaller diameter threshold. Patients undergoing a valve replacement or repair may undergo concomitant aortic resection if the aorta measures 45 mm or greater, which is validated as safe [35]. These guidelines for prophylactic aortic replacement are governed by aortic diameter, which is insufficient to predict acute aortic events for BAV aortopathy [15]. The need for biomarkers and advanced imaging to refine surgery timing is well-established [36,37].

### 4.6. Adolescent Transition Clinic

Longitudinal studies of congenital heart disease are limited by the inability to connect pediatric disease progression with adult outcomes. Through the creation of a dedicated, adolescent transition clinic, we have established a single clinical registry for pediatric and adult patients with a unified data dictionary. A dedicated social worker is also embedded in the clinic to help assess and promote transition readiness and to help families navigate insurance changes across young adulthood. Though not every pediatric patient is ultimately funneled through the transition clinic, many of the most severe cases or advanced phenotypes are concentrated in this multidisciplinary clinic. Compared to another large institutional cohort of pediatric patients with isolated BAV in which 7% of subjects had moderate aortic regurgitation or greater and the median ascending aorta Z score was + 2.3 [38], our transition clinic represents an enriched population with nearly 40% of patients having important valve disease or aortopathy. Given that loss to follow up in the adult congenital heart disease population is associated with male sex, moderate disease complexity, fewer pediatric interventions, a gap in insurance coverage or lack of a transition program [39,40], and that lapses in care increase the risk of urgent cardiac intervention [41], the adolescent BAV transition clinic addresses an important gap for an “at risk” population. Similarly, this collaboration has facilitated screening of FDR across all age ranges, including fetal counseling.

### 4.7. Translational Research Participation

Aortic diameter and growth rate are only surrogates for the risk of acute aortic syndromes, yet they underlie contemporary surgical decision-making in BAV aortopathy [36,42]. Moreover, considerable variability in surgical management can be attributed to differing perceptions of the role BAV-mediated hemodynamics play in the expression of ascending aortopathy [43].

To address this unmet need for noninvasive biomarkers that more closely reflect aortic wall integrity on a patient-by-patient basis, we used 4D flow CMR (Figure 2) in BAV patients prior to surgery to quantify wall shear stress (WSS)—a hemodynamic parameter known to influence aortic wall remodeling in animal models [44,45,46]. Patient tissue that was collected intraoperatively was compared within each patient’s aorta for histopathology, molecular markers of aortopathy and biomechanical testing. This unique methodology enabled patients to serve as their own internal controls. We documented that, compared to regions subjected to normal WSS, adjacent regions in each aorta subjected to elevated WSS exhibited greater quantitative histopathology and molecular markers of aortopathy, as well as differences in biomechanical testing [19]. Furthermore, we found that the magnitude of elevated WSS directly correlated with aortopathy expression in the tissue, in addition to differences in valve phenotype (AS vs. AI) and ascending aortic diameter [47].

This work shows that noninvasive hemodynamic biomarkers can be mapped in the aortas of human patients and corresponds to the location and severity of disease. With further validation, mapping diseased tissue using this technology could allow surgeons to strategize what tissue should be removed, as well as what tissue is healthy and should be left in individual patients; they can be as aggressive or conservative as needed for individualized patient treatment.

## 5. Conclusions

The clinical and translational research needs of the BAV patient population warrant a clinical model which supports the patient-family with BAV and promotes translational efforts that may refine surgical timing for subsequent generations. Dedicated clinical support for BAV family screening can help detect first-degree relatives with BAV or aortopathy. Patients can be safely followed by a multidisciplinary team to detect indications for surgery and provide comprehensive care to the entire family with BAV.

## Figures and Tables

**Table 1:** Baseline characteristics of patients presenting to bicuspid aortic valve (BAV) clinic, by clinical demographics.

Variable	*n*	Entire Cohort (*n* = 887)		Medical (*n* = 455)		Surgical (*n* = 388)		Medical to Surgical (*n* = 44)		*p*-Value
Age	887	52.0	±14.5	46.0	±13.6	58.9	±12.6	54.3	±11.7	<0.001
Maximum aortic diameter (mm)	636	42.1	±6.4	40.7	±5.2	43.9	±7.3	43.4	±4.5	0.001
Gender (female)	887	233	(26%)	156	(34%)	73	(19%)	4	(9%)	<0.001
Family History BAV	704	94	(13%)	64	(14%)	25	(11%)	5	(12%)	0.52
Family History of Ascending Aortic Aneurysm	859	19	(2%)	16	(4%)	3	(1%)	0	(0%)	0.015
Sievers Fusion Pattern	782									0.001
Type 0										
Aneroposterior		27	(3%)	12	(3%)	12	(3%)	3	(8%)	
Lateral		24	(3%)	8	(2%)	14	(4%)	2	(5%)	
Type 1										
Right-Left Coronary		574	(73%)	308	(77%)	245	(71%)	21	(53%)	
Right-Noncoronary		124	(16%)	61	(15%)	54	(16%)	9	(23%)	
Left-Noncoronary		9	(1%)	6	(2%)	2	(1%)	1	(3%)	
Type 2 (Unicuspid)		24	(3%)	3	(1%)	17	(5%)	4	(10%)	

**Table 2:** Characteristics of surgery group by procedure.

Variable	*n*	Entire Cohort (*n* = 399)		SAVR only (*n* = 224)		SAVR + AN (*n* = 163)		TAVR (*n* = 12)		*p*-Value
Age	399	59.5	±12.0	60.9	±11.6	56.4	±11.2	76.2	±9.7	<0.001
Maximum Aortic Diameter (mm)	388 (213, 163, 12)	42.9	±6.9	38.6	±4.5	48.9	±4.7	38.3	±4.7	<0.001
Gender (female)	399	95	(23.8%)	64	(28.6%)	27	(16.6%)	4	(33.3%)	0.017
Pure AI	399	46	(11.5%)	15	(6.7%)	31	(19.0%)	0	(0.0%)	<0.001
Bioprosthetic valve	399	364	(91.2%)	215	(96.0%)	149	(91.4%)	0	(0.0%)	<0.001
30-Day Mortality	399	3	(0.8%)	1	(0.4%)	2	(1.2%)	0	(0.0%)	0.649
Free from Aortic Valve Reoperation	290 (172, 112, 6)	284	(97.9%)	167	(97.1%)	111	(99.1%)	6	(100%)	0.475
Free from Late Aortic Valve-in-valve Procedure	290 (172, 112, 6)	289	(99.7%)	171	(99.4%)	112	(100%)	6	(100%)	0.709
Free from Late Aortic Intervention	290 (172, 112, 6)	289	(99.7%)	172	(100%)	111	(99.1%)	6	(100%)	0.451

Excludes patients with valve sparing root replacement (*n* = 15) and aortic valve repair (*n* = 18).

**Table 3:** Baseline Characteristics of Patients Presenting to BAV Bridge Transition Clinic.

Variable	*n*	Entire Cohort	
		(*n* = 45)	
Age (years)	45	19.6	± 2.8
Max aortic diameter (mm)	39	31.8	± 6.6
Aortic diameter Z score > 4	38	7	18.4%
Gender, Male	45	36	80.0%
Family history of BAV	40	9	22.5%
Family history of thoracic aneurysm	40	3	7.5%
History of aortic coarctation	44	3	6.8%
History of infective endocarditis	44	0	0.0%
History of aortic valve procedure	44	2	4.5%
History of congenital surgery/procedure	44	2	4.5%
Aortic Stenosis	41		
Mild		8	19.5%
Yes		4	9.8%
Aortic Insufficiency	41		
Trivial		8	19.5%
Mild		13	31.7%
Moderate		5	12.2%
Severe		1	2.4%
None		14	35.0%
Fusion Pattern	42		
LAT- No Raphe		1	2.4%
Right-Left (R-L)		18	42.9%
Right-Noncoronary (R-N)		3	7.1%
RL-RN		1	2.4%
Undetermined		19	45.2%

## Supplementary Materials

Supplementary File 1
